# Development and clinical implementation of an MRI‐only planning workflow featuring deep learning‐based synthetic CT for prostate cancer external beam radiotherapy

**DOI:** 10.1002/acm2.70228

**Published:** 2025-08-21

**Authors:** Jie Deng, Xiao Liang, Viktor Iakovenko, Boyu Meng, Junjie Wu, Weiguo Lu, Raquibul Hannan, Neil Desai, Daniel Yang, Aurelie Garant, Yang Kyun Park, Mu‐Han Lin, Steve Jiang, Andrew Godley

**Affiliations:** ^1^ Department of Radiation Oncology University of Texas Southwestern Medical Center Texas USA; ^2^ Department of Radiation Medicine & Applied Sciences, Medical Physics Division University of California San Diego La Jolla California USA

**Keywords:** external beam radiation therapy, MRI‐only planning, prostate cancer

## Abstract

**Background:**

In external beam radiation therapy (EBRT) for prostate cancer, both MRI and CT are typically used—CT provides electron density for dose calculation and visualization of bony anatomy and fiducial markers, while MRI offers superior soft tissue contrast. With recent advances in deep learning, synthetic CT (sCT) images can now be generated from MRI, potentially eliminating the need for separate CT scans.

**Purpose:**

To clinically implement an MRI‐only planning (MROP) workflow for both X‐ray and MRI‐guided systems.

**Methods:**

MROP implementation involved optimizing MRI simulation protocols, developing a deep learning‐based sCT generation method, and automating fiducial marker detection. A multichannel CycleGAN model was trained using T1‐weighted Dixon images to generate sCT. For SBRT cases, the immobilization device was auto‐inserted into sCT images. Fiducial markers were detected using a quantitative susceptibility mapping (QSM) method. Validation included retrospective comparison of Hounsfield units and dosimetric plans to those based on planning CT for 11 patients, followed by prospective evaluation of ten patients.

**Results:**

The MRI protocol included T2‐weighted 3D imaging for contouring, T1‐weighted Dixon for sCT, and T2*‐weighted GRE for fiducial detection. The MROP workflow was prospectively applied in 10 patients (three SBRT, seven conventional). Dosimetric comparisons showed <1% difference between MROP and CT‐based plans across all PTVs. The QSM‐based fiducial detection achieved a 95.5% success rate (63/66 markers) and reduced detection time from ∼10 to 2 min per patient. All MROP components were integrated into the treatment planning system via scripting. Implementation reduced simulation scheduling time by 7.1 days.

**Conclusion:**

We successfully implemented an MROP workflow for prostate cancer EBRT, eliminating the need for CT, reducing radiation exposure, and improving clinical efficiency. Over 500 patients have been treated to date using this approach on both C‐arm and MR‐guided systems.

## 1 INTRODUCTION

Traditional radiation therapy (RT) requires generating a treatment plan based on computer tomography (CT) alone or in combination with magnetic resonance imaging (MRI) scans prior to treatment. This process, known as simulation, ensures accurate delineation of the target and surrounding critical organs.^1^ Additionally, it establishes patient positioning and immobilization. CT remains the primary imaging modality in external beam radiation therapy (EBRT) planning for pelvic cancer,^2^ as it provides electron density (ED) information for dose calculation and serves as the reference for X‐ray‐based image‐guided radiotherapy (IGRT).^3^ However, CT is limited in tumor‐to‐tissue contrast due to its reliance on X‐ray linear attenuation and its susceptibility to image artifacts. Studies have reported that CT‐only simulation can overestimate prostate volume by 30%–40% and introduce more variability in target delineation than daily setup uncertainties.^4^ To compensate for these uncertainties, large margins (5–10 mm) are typically required, increasing the risk of toxicity to the surrounding healthy tissues. MRI is widely recognized for its superior soft tissue contrast for target and normal tissue delineation, which significantly reduces intra‐ and inter‐observer contouring variability when incorporated in treatment planning for prostate cancer.^5^, ^6^ Commonly, a multi‐modality simulation workflow is employed, where MRI serves as a secondary imaging modality to enhance anatomical visualization and segmentation for treatment planning.^7^, ^8^ It must be co‐registered with CT to obtain the electron density information necessary for dosimetry plan generation. However, this combined workflow adds complexity to patient scheduling and places additional demands on simulation resources. Furthermore, anatomical and positioning variations between CT and MR simulation acquisitions can introduce systematic errors during dataset registration, leading to uncertainties of 2–3 mm in prostate cancer RT.^9^, ^10^ These uncertainties are further compounded by inconsistent bowel and bladder filling between the two scans—typically performed 30–60 min apart, which can significantly alter organ shape and position.

To overcome these limitations, an MR‐only treatment planning (MROP) approach has been proposed to eliminate the need for CT simulation, thereby reducing imaging‐related radiation exposure, eliminating systematic CT‐MRI registration errors, optimizing the use of departmental resources, and improving patient convenience.^11^, ^12^ Since its introduction in 2016, it has gained attention from radiation oncology centers, with several institutions pioneering its implementation.^13^ A study published in 2017 detailed the implementation of an MROP for prostate cancer simulation and planning, highlighting its feasibility and benefits, but still required an x‐ray image for identifying fiducials.^14^ They later summarized their experience with a total of 585 prostate cancer patients who underwent an MR‐only simulation and planning between 2016 and 2018 and demonstrated that MROP was successfully implemented for the majority of patients in the clinic.^15^ They also noted that MR‐CT or CT‐only pathway may still be required for patients with MR contraindications. An additional center reported the successful implementation of an MRI‐only radiotherapy planning program, utilizing two dedicated MRI platforms (1.5 and 3.0 T) for EBRT across various treatment sites.^16^ These previous studies were limited by the field of view of the sCT.

Although MRI provides a variety of image contrasts to differentiate tissue types, it lacks the ED information necessary for radiation dose calculation.^17^ The development of MRI‐based synthetic CT (sCT) is a critical component of MROP, enabling accurate dose calculations. Various techniques for sCT generation have been widely studied.^18^ A commercially available software package, MR for calculating attenuation (MRCAT, Philips Healthcare, the Netherlands) uses the water and fat signal separation from Dixon MRI sequences to facilitate sCT generation.^19^ However, MRCAT has limitations, including fixed acquisition parameters and restricted anatomical coverage. In particular, MRCAT has a maximum length of 24 cm, insufficient for treating patients with nodal involvement. Around 30%–40% of prostate patients treated with external beam radiation therapy require or elect for nodal treatment at our institution. The number of elective nodal irradiations has increased since the POP‐RT trial.^20^ Recent advancements in deep learning‐based sCT generation algorithms have significantly improved the accuracy of sCT images, paving the way for broader MROP adoption.^21^, ^22^, ^23^ In our work, we developed an in‐house sCT model using an unsupervised CycleGAN approach to improve the reliability of bony structures in sCT images with improved accuracy in both bone and soft tissue representation over a large field of view.^24^

For prostate cancer treatment on conventional linear accelerators (Linacs) equipped with onboard kV/MV X‐ray‐based IGRT, localization of intra‐prostatic gold fiducial markers (FMs) is often required to aid in target localization.^25^, ^26^, ^27^ These markers, implanted prior to CT/MRI simulation, appear as bright signals on CT; however, their visualization on MRI can be challenging. The appearance of signal voids due to susceptibility effects can be confused with air pockets and calcifications, making FM detection highly subjective and dependent on imaging sequences and parameters.^28^ Several studies have explored automated or semi‐automated MR‐based gold FM detection methods, aiming to improve accuracy and reliability in FM localize on MRI.^29^, ^30^ We implemented a QSM sequence to highlight FM in MR.

With the invention and increasing clinical adoption of MR‐linear accelerator (MRL) systems, MRI‐guided RT (MRIgRT) offers significant improvements in tumor control while minimizing damage to surrounding healthy tissues compared to X‐ray‐based IGRT.^31^ The superior soft‐tissue contrast provided by online MRI enhances visualization of the target volume and organs‐at‐risk (OARs), enabling high‐precision treatment for prostate cancer. Daily MR images are aligned with MRI simulation images without the need for FMs. MRIgRT facilitates online adaptive re‐planning, thereby enhancing the accuracy of treatment delivery.^32^, ^33^, ^34^, ^35^ The benefits of MROP are maximized in the context of MRIgRT, providing an even‐stronger rationale for omitting CT simulation in this clinical workflow.

In this study, we demonstrated the process of establishing a comprehensive cross‐platform MROP workflow that integrates both MRIgRT and C‐arm‐based IGRT for prostate treatment. This process includes optimizing MRI simulation protocols and procedures, developing sCT generation and FM detection methods, conducting validation studies, and implementing the program clinically. This workflow is designed to be adaptable and can be expanded to additional body sites beyond prostate cancer. This paper provides guidance for other centers looking to establish their own MROP programs and is the first to present a combined approach for MR and CT based linacs.

## 2 METHODS

### 2.1 Workflow design

The workflow design for MROP differs between cone‐beam CT (CBCT)‐guided C‐arm Linac and MRL systems, particularly in patient setup and image registration between simulation and online images, where sCT‐to‐CBCT is used for C‐arm systems, while MR‐to‐MR is employed for MRL. Successful implementation requires proactive collaboration among radiation oncologists, medical physicists, and radiation therapists to define resources and procedures. Early‐phase physician input is essential to establish clinical priorities, identify key outcomes, and determine the target body sites for implementation. Support from R&D teams and vendors is needed for software and hardware integration, along with IT and programmer assistance for seamless data transfer and automation. Additionally, efficient scheduling and staffing are necessary to accommodate workflow changes effectively.

### 2.2 MR simulation imaging protocol

The MR simulation was conducted on a Philips Ingenia Ambition 1.5T MRI scanner (Philips Healthcare, the Netherlands). The MR‐Sim protocol was designed to acquire various imaging for treatment planning, including T2‐weighted 3D images for organ delineation and contouring, T1‐weighted 3D Dixon images with separated water, fat, and in‐phase images for sCT generation, and multi‐echo T2*‐weighted images for FM detection. Table 1 summarizes the MR simulation protocol with detailed imaging parameters and acquisition times. Slice thickness was 2 mm for stereotactic body RT (SBRT) and 3 mm for non‐SBRT protocols. Additionally, CT simulation was performed using a Philips Brilliant CT Big Bore scanner following the departmental protocol, 120 kVp and same slice thicknesses as above. Quality Assurance (QA) for the MR simulator was conducted in accordance with ACR certification requirements and TG‐284 recommendations.^36^

**TABLE 1:** MRI simulation imaging protocol for MROP. FOV: Field‐of‐view; SI: Superior‐inferior; TR: Repetition time; TE: Echo time; TSE: Turbo spin echo; GRE: Gradient echo; TFE: Turbo field echo; NSA: Number of signal acquisition; SENSE: Sensitivity encoding; CS: Compress sensing; WFS: Water fat shift; IP: In‐phase.

Sequence type	Purpose	FOV × SI coverage (mm)	Pixel size × slice thickness/gap (mm)	Other parameters	Scan time (min: s)
Organ check	Bladder& rectum check	400 × 400 × 208	1.8 × 2.2 × 20		0:42
T2 TSE 3D	Contouring	450 × 450 × 400	1.3 × 1.3 × 1.35/0	TR = 1250 ms, TE = 184 ms, CS‐SENSE = 10, TSE factor = 136, NSA = 2, WFS = 0.175 pixel	3:38
T1 GRE mDixon 3D Vane (water and IP)	Treatment planning (SBRT)	450 × 450 × 400	1.75 × 1.75 × 2.0/0	TR = 5.6 ms, TE = 1.8/3.5 ms, SENSE = 2, TFE factor = 112, NSA = 1, WFS = 0.3 pixel	5:21
T1 GRE mDixon 3D Vane (water and IP)	Treatment planning (non‐SBRT)	520 × 520 × 400	1.75 × 1.75 × 3.0/0	TR = 5.6 ms, TE = 1.8/3.5 ms, SENSE = 2.5, TFE factor = 99, NSA = 1, WFS = 0.3 pixel	4:08
T2* 3D GRE 6‐echo (magnitude/phase)	Internal marker for fiducial detection	180 × 180 × 90	1.0 × 1.0 × 2.0/−1.0	Echoes = 6, TR = 28 ms, TE = 3.1‐24.6 ms,ΔTE = 4.3 ms, flip angle = 15°, SENSE = 3, NSA = 1	3:16
Post T1 GRE mDixon 3D Vane	Same as T1 GRE mDixon				

### 2.3 MRI simulation procedure

#### 2.3.1 SBRT frame and coordinate conversion

In SBRT, a frame is used to ensure patient positioning accuracy. The SBRT frame includes coordinate marks that are visible on CT, allowing the derivation of frame coordinates for determining or confirming the treatment position. However, neither the frame nor the marks are visible on MRI. We modified the original SBRT frame to add an MR‐visible coordinate system using three silicone rods embedded at the bottom of the frame. By reconstructing the 3D coordinates of these rods, we can convert their MRI positions to the SBRT frame coordinates for any point of interest on MRI based on its spatial relationship with the coordinate rods. The visibility of the rods on MR image also facilitated accurate placement of the frame in the sCT image generated from the MRI. This marking strategy is generalizable and can be applied to other types of immobilization devices using a similar approach.

#### 2.3.2 Patient setup

**FIGURE 1:**
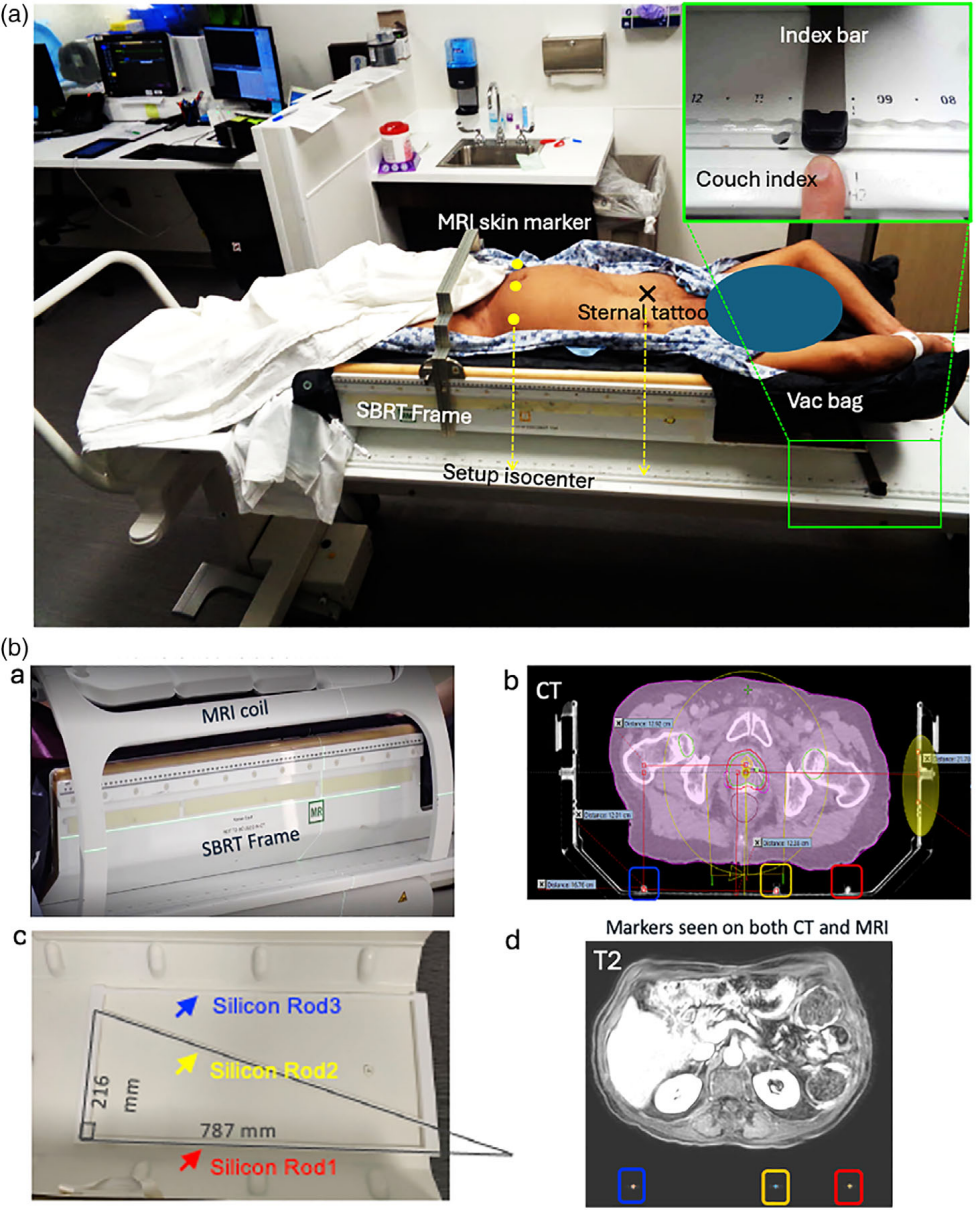
The MRI simulation procedure for the prostate SBRT setup (a) and the modified frame system with the MRI Coil (b.a), the built‐in silicon rods for index conversion (b.c), the T2w image with detected fiducial rods (b.d), and the sCT with inserted SBRT frame and corresponding silicon rods used to align with the SBRT frame index (b.b).

Patients receive instructions of our standard‐of‐care protocol, arriving with a full bladder and an empty rectum. Bladder filling status is assessed before simulation using a handheld bladder scanner (BladderScan BVI 6100, Verathon Inc.). The MRI simulation is conducted on a flat tabletop customized with an MRL couch index (Philips Healthcare). The MR simulator is housed within our Radiation Oncology Department and staffed by both a MR certified technologist and a Radiation Therapy certified technologist, familiar with simulation procedures and immobilization equipment. For immobilization, Flex Lock (CQ Medical) is used for non‐SBRT setups, while a vacuum bag within the SBRT frame is used for SBRT patients. Patients are initially positioned on the MRI tabletop outside the scanner room (Zone III). The index bar at the top of the frame is used to align and center the frame on tabletop. Setup photos are taken, and sternal and pelvic tattoos are applied for positioning the patient in the frame if it is used (SBRT) before transferring the MRI table into the scanner room. Inside the scanner room, an external laser system is used to straighten the patient and to assist in placing three MR‐visible vitamin E markers on the patient's surface at the approximate level of the prostate to mark the setup isocenter. The corresponding MRL couch indexes for the index bar of SBRT frame, the setup isocenter, and the sternal tattoo are recorded separately in the simulation documentation. The MRI simulation procedure for the SBRT setup and the modified frame system is illustrated in Figure 1.

### 2.4 Synthetic CT generation algorithm

We modified the original CycleGAN model,^37^ which has been used for MR‐to‐sCT conversion in an unsupervised training manner, by incorporating two input channels with different MR images (fat and IP) reconstructed from the T1 Dixon sequence for sCT generation. To enhance the accuracy of bony structures in sCT images, we added bony structure constraints into the loss function. Details on the model development and evaluation have been published previously.^24^ 20 prostate cancer patients were used for training and 11 for testing. Using an Nvidia Tesla V100‐SXM2 GPU, the execution time is around one second per slice. We demonstrated that multi‐channel CycleGAN with bony structure constraints achieved the lowest mean absolute error both within the bone and whole body, as well as the highest Dice similarity coefficient of all bony structures compared to the planning CT. Our sCT has SI coverage of 400 mm and the transverse field of view of 550 × 550 mm^2^, allowing us to include patients with nodal involvement.

### 2.5 FM detection algorithm

Magnetic susceptibility can serve as a contrast source to differentiate implanted gold markers from soft tissue, computed from the magnitude and phase images of a multi‐echo T2*‐weighted gradient echo (GRE) sequence. The prostate contour was first generated on The T2w images using an in‐house developed auto‐segmentation tool to restrict the search region for the quantitative susceptibility mapping (QSM) algorithm. Specifically, nonlinear fitting was performed on the complex GRE images to generate the total field map, followed by Laplacian phase unwrapping and background field removal via projection onto the dipole field to obtain the local field map. The QSM map was then generated through local B0 field to susceptibility inversion using a background zero‐referencing method (MEDI+0). Subsequently, image processing steps, including histogram analysis, removal of discrete pixels, detection of slice‐connected regions, and localization of each region's centroid, were implemented to identify each gold FM. Detection efficiency and location accuracy were tested in a cohort of 22 patients, each with three gold FMs of ∼0.3 mm diameter (Gold Anchor™, Naslund Medical AB) implanted in the prostate prior to MRI/CT simulation. Note that the parameters in the QSM algorithm should be fine‐tuned to optimize the detection of the specific gold FMs used. QSM‐based detection efficiency was compared with manual detection performed by an experienced medical physicist, while detection accuracy was assessed using CT as the reference. The detection rate was calculated as the ratio of successfully detected FMs to the total number of FMs in the cohort. Detection accuracy was determined by measuring the distance between QSM‐identified FMs and those identified on CT scans, considering only successfully detected FMss. Additionally, we evaluated the clinical benefit of the QSM‐based method in assisting clinicians with FM detection. Two weeks after the medical physicist completed the initial round of manual FM detection on GRE images for the 22 patient cases, the detection process was repeated with access to QSM‐identified FMs. We then compared the detection rate, and the average time spent per patient between the first round of manual detection and the QSM‐assisted detection to assess its effectiveness as a tool for FM identification.

### 2.6 Retrospective evaluation

Five prostate cancer patients who received EBRT on C‐arm Linacs were selected for the retrospective study. To assess bony structure visualization and HU accuracy, CT simulation images were rigidly registered to MRI simulation images, then deformed to match the MRI and used as the reference standard. For dosimetric plan comparison, the reference CT and sCT were rigidly registered. The clinically optimized plan, originally developed based on the reference CT was directly applied to the sCT, followed by dose recalculation. The dose distributions for the planning target volume (PTV) and OARs from the reference CT‐based plan and the sCT‐based plan were then compared.

### 2.7 Workflow integration

We integrate both sCT generation and QSM‐based FM detection into the Eclipse (treatment planning system (TPS) Varian, Palo Alto, CA) via the Eclipse Scripting Application Programming Interfaces (ESAPI). The script retrieves DICOM files of the source MR images from the server, executes the image processing code, and imports the resulting sCT with structure sets of FMs back into Eclipse for treatment planning. Additionally, a script was developed for inserting the patient immobilization SBRT frame structure set onto the MRI image or sCT. Users manually align the frame structure with the visible bright coordinate rod signals on T2‐weighted MR images, and once inserted, the structure is automatically copied to the sCT image set.

### 2.8 Prospective evaluation

**FIGURE 2:**
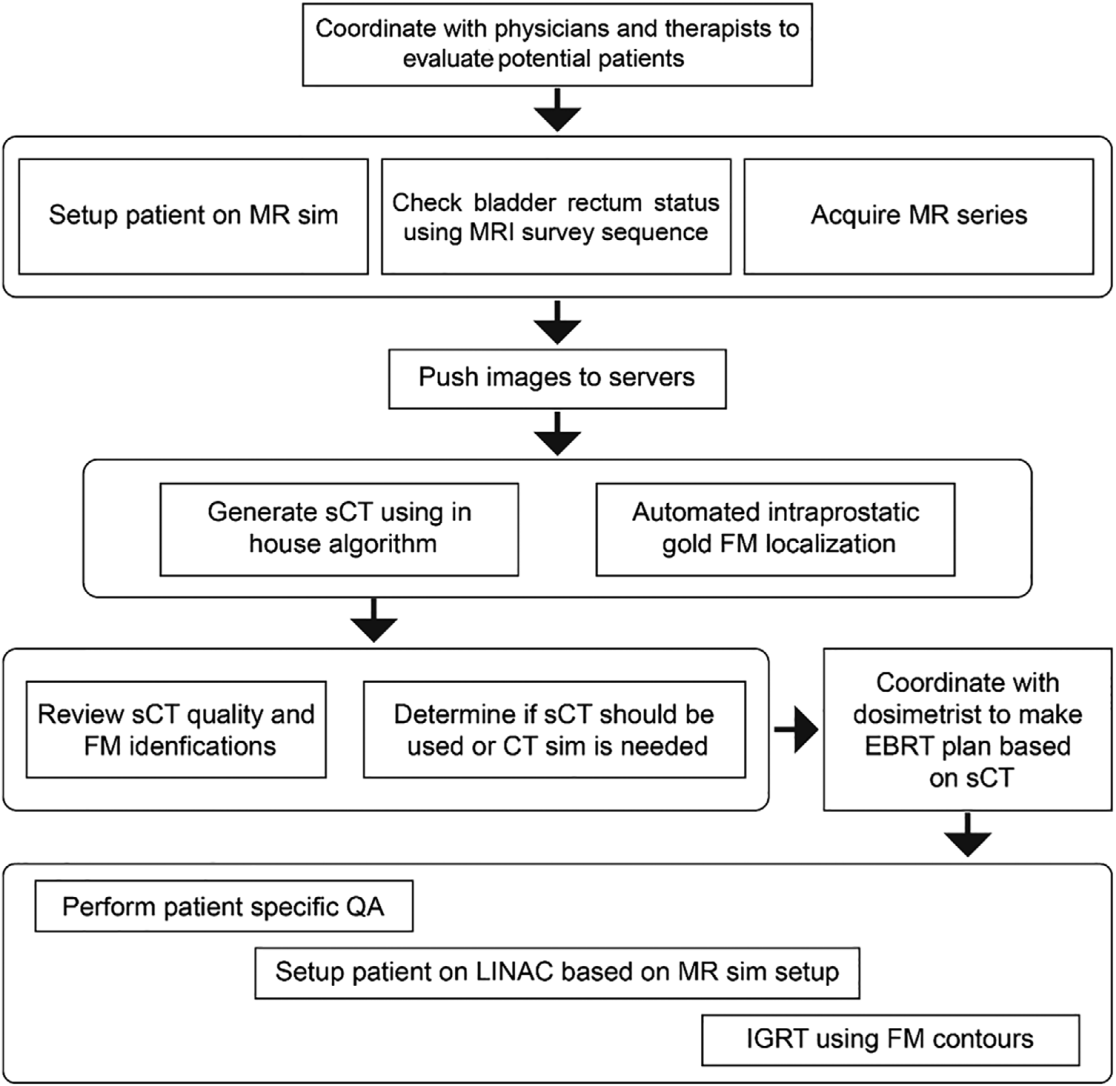
MROP workflow. For the MR‐Linac, the FM do not need to be identified, and the IGRT is performed using the MR directly.

The MROP workflow was tested in a cohort of prostate cancer patients (*N* = 12) receiving EBRT, selected by the physician for prospective evaluation. The entire prospective evaluation workflow is summarized in Figure 2. At the time of simulation, patients had undergone a prior appointment for internal gold FM and spacer gel placement (SpacerOAR, Augmenix, Inc.). MR technologists evaluated each patient against exclusion criteria for MROP, including hip prosthesis, implants or devices in the abdomen or pelvis, excessive gas, or a body circumference at the iliac crest level exceeding 49 inches (measured on previous diagnostic imaging or at consult). Approximately 5% of patients screened for prostate cancer have a hip implant and would be excluded.^38^The technologists then consulted the physics team to determine whether a CT simulation was necessary. A quick organ check MR scan was taken for the physician to confirm bladder fill and rectum empty before acquiring the planning sequences and confirm no artifacts. This step was added after an early prospective case had poor bowel preparation. As with CT simulation, imaging will not proceed with incorrect bladder or bowel filling, and patient and staff will have to rectify this. In cases where incidental metal artifacts are found near the treatment region (e.g., prostate or prostate plus nodes) that could interfere with image quality or treatment planning, an additional CT scan will also be ordered. Once all MR images were transferred to the treatment planning system (Eclipse, Varian, Palo Alto CA), scripts were executed to generate the sCT and locate and contour the FMs. A physicist reviewed the quality of the sCT and the accuracy of FM contours to confirm its suitability for treatment planning. The T1 Dixon water images, used as the source for sCT generation, served as a cross‐reference with T2*‐weighted GRE images to verify FM location, as gold FMs appear as signal voids compared to prostate tissues. It is possible for the anatomy to shift between acquisition of MR sequences due to bladder continuing to fill, or movement of rectal gas. If slight discrepancies in FM locations were observed between the sequences, their locations were adjusted to align with the signal voids in the T1 water images. Once finalized, the FM contours were transferred onto the T2w 3D images. The physician then delineated the target volumes and OARs on the T2w 3D images.

To ensure no delays in treatment in case of MROP failure during the prospective evaluation phase, a CT scan was performed after MR simulation to be available for planning if necessary. For patients planned for treatment on C‐arm Linac, all contours were transferred to the sCT, as it shares the same coordinate system with MR images. The dosimetrist then proceeded with treatment planning on the sCT dataset. For patients planned for MRL treatment, sCT is used for bulk ED calculation, both T2w 3D MRI and sCT was transferred to Monaco TPS for treatment planning. To assess dosimetric accuracy of the MROP workflow with sCT, the backup CT simulation images were rigidly registered to the MR images, and MROP clinical treatment plans were recalculated on the simulation CT images.

## 3 RESULTS

### 3.1 MRI simulation images

**FIGURE 3:**
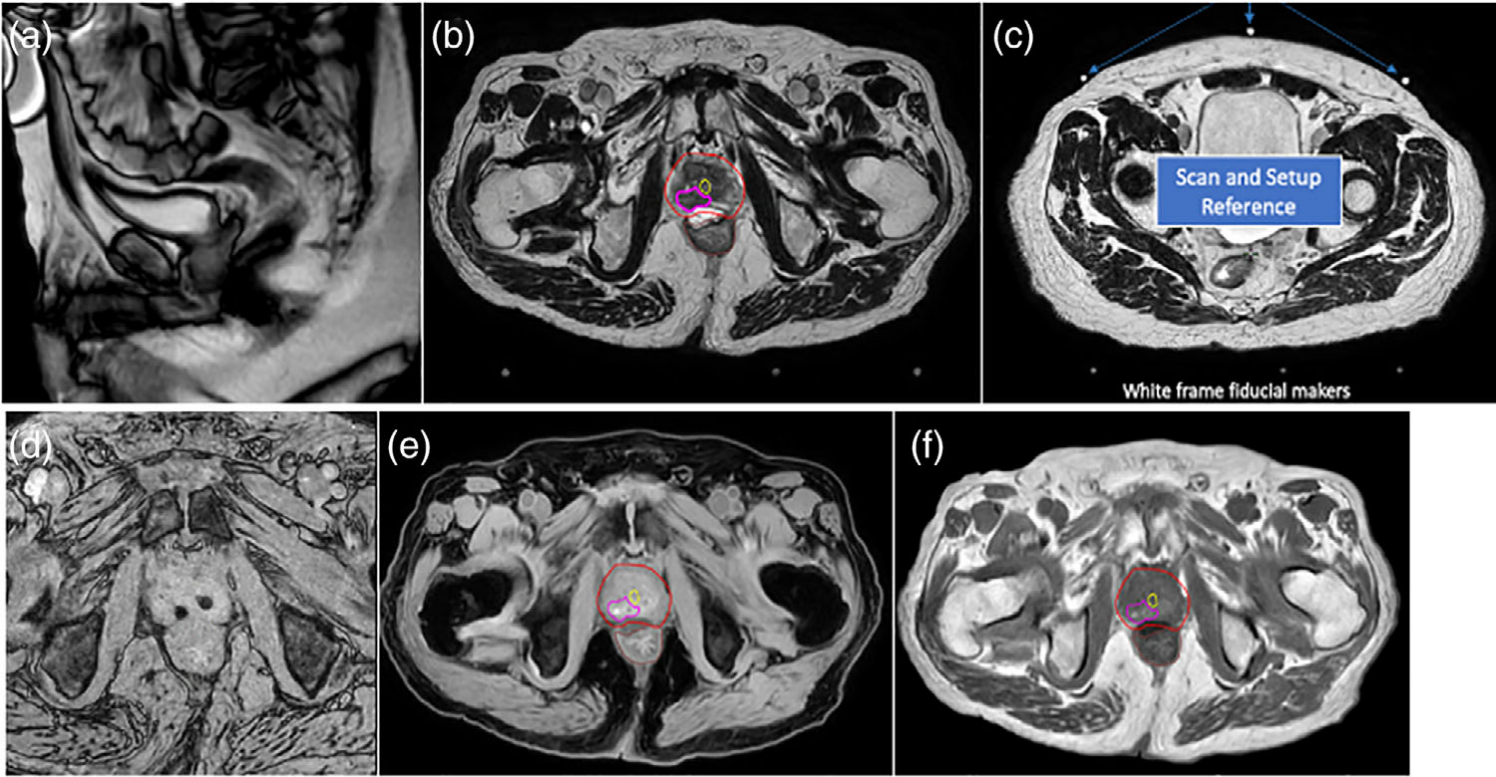
A patient example of MRI simulation images. (a) Organ‐check images indicating suboptimal bladder filling, requiring further preparation. (b) T2w 3D images with target and OAR contours. (c) T2w 3D images showing bright MRI markers on the skin and fiducial rods at the bottom of the SBRT frame. (d) Internal marker images depicting gold fiducial markers as signal voids. (e, f) T1w water and IP images reconstructed from T1w Dixon images, used for sCT generation.

A patient example of MRI simulation images is shown in Figure 3. These include organ check images to assess the bladder filling status and the presence of gas in the rectum. T2w 3D images are used not only for contouring the target and OARs but also for frame insertion into the images. Three MRI markers on the skin and coordinate rods at the bottom of the SBRT frame appear bright, serving as references for isocenter setup and frame insertion alignment, respectively. Internal marker images acquired using the T2*w GRE sequence depicted gold FMs as signal voids larger than their actual size due to susceptibility effects. T1w water images exhibited hyperintense signals in non‐fat soft tissues but appeared hypointense in fat, air, and bone, whereas T1w IP images showed hyperintensity of fat and bone tissue. Both T1w water and IP images were reconstructed from T1w Dixon images are used to generate the sCT. These images are inherently co‐registered; however, in rare cases, organ misalignment may occur due to changes in bladder filling or rectum shape during the 20‐min MRI exam, leading to a shift in prostate position, requiring manual co‐registration to realign the images. To perform this registration, the DICOM coordinate link between the images must first be broken. Registration is then performed firstly between the QSM sequence where the FMs are most visible and identifiable as metal, and the T1 sequence, where the FMs are still visible. Once the location of the FM is confirmed in the T1 sequence, the T1 is then registered to the T2 sequence using both the anatomy and the FMs. With the help of the T1, the voids in the T2 representing the FMs can be identified. This leaves the T2 as the sequence for planning, with both the organ and target contours, and the true FMs identified for alignment at the time of treatment. This procedure has been required only 5 times since the start or MROP at our institute.

### 3.2 Automatic fiducial marker localization

**FIGURE 4:**
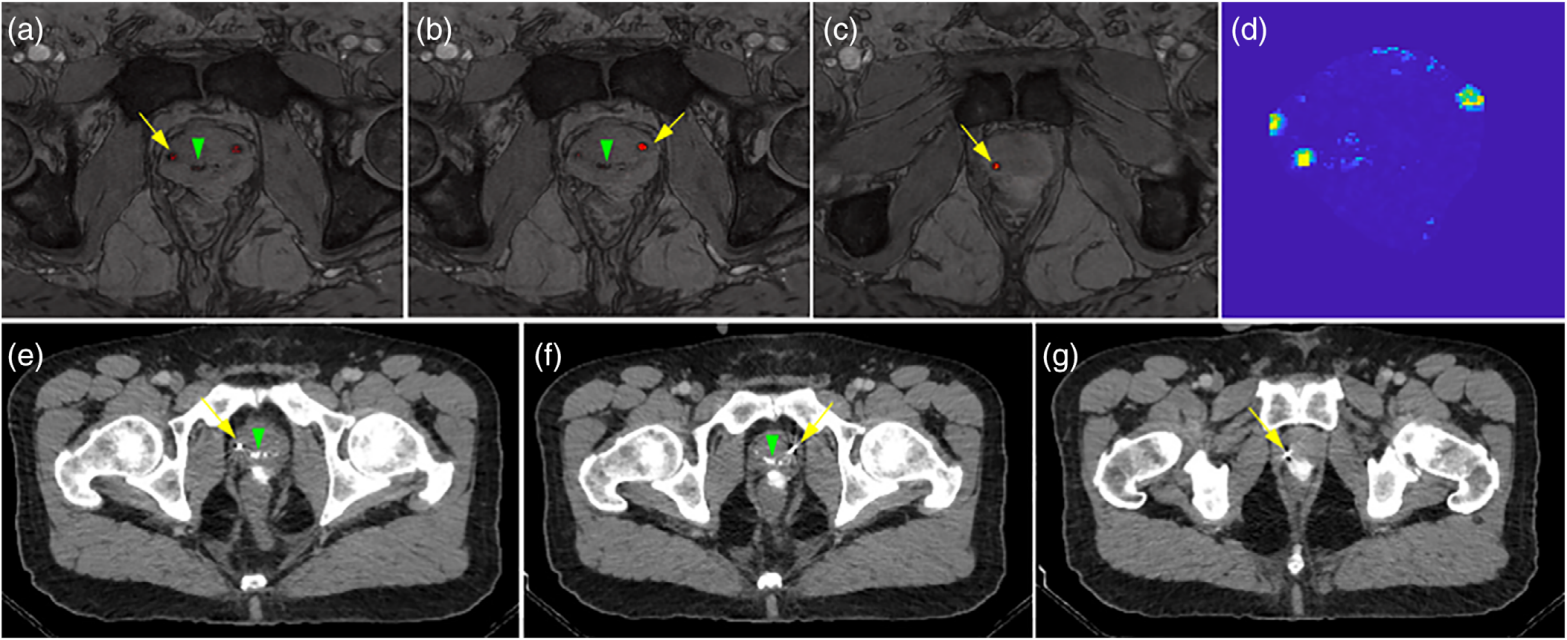
An example illustrating the performance of QSM‐based fiducial marker detection using multi‐echo T2*w MRI images (a–c) compared with corresponding CT‐based detection (e–g). Yellow arrows indicate the location of each fiducial marker. The green arrowhead points to a calcification, which was excluded by the QSM method, while the three gold fiducial markers appear bright on the QSM map (d).

In 22 patients with 66 FMs, the detection rates using QSM and manual identification by the experienced physicist were 89.4% and 83.3%, respectively. The QSM auto‐detected FM locations were displaced by an average of 2.6 mm from the CT FM locations, which was comparable to the accuracy of manual detection by the medical physicist. The physicist re‐evaluated the images 2 weeks later with access to the QSM‐generated FM locations. With this additional information, the detection rate increased to 95.5% (63 out of 66 FMs), and the average detection time per patient was reduced from ∼10 to 2 min. The 3 FMs not detected were at the very edge of the prostate. For detection and to prevent migration, FMs must be placed following standard guidelines.^39^ Examples illustrating the performance of QSM‐based detection using multi‐echo T2*‐weighted MRI images compared with CT‐based detection are shown in Figure 4. The proposed QSM method effectively distinguished FMs from calcifications and air pockets, as the magnetic susceptibility of gold FMs (−36 ± 4 ppm) differs from air (+0.024 ppm) and calcification (−21 ± 3 ppm).^40^

### 3.3 Retrospective evaluation

**FIGURE 5:**
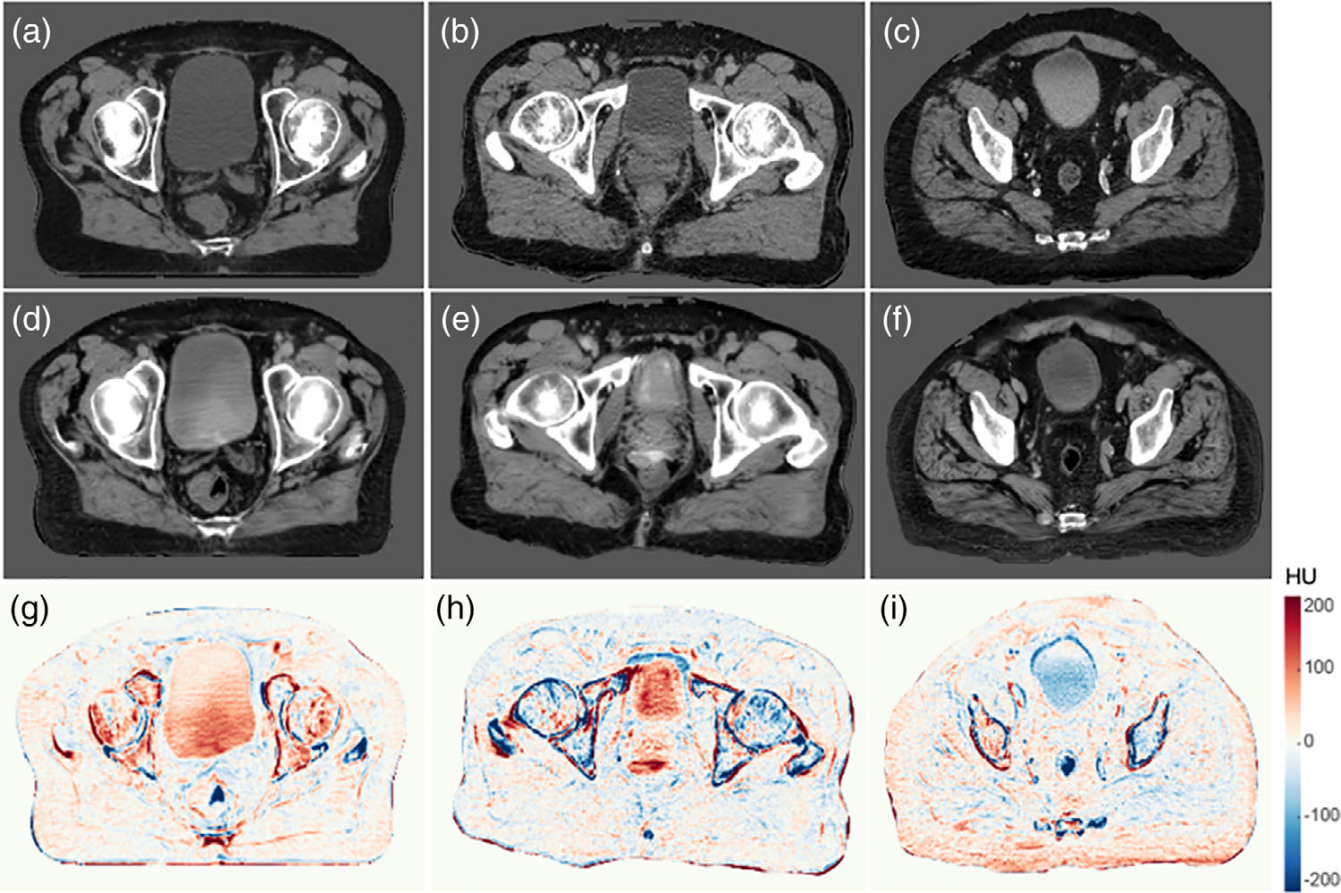
sCT quality assessment. (a–c): Axial view CT images of 3 different patients. (d–f): Corresponding sCT images. (g–i): Difference in HU between CT and sCT.

The retrospective analysis results have been published in our previous study.^24^ In summary, the multi‐channel CycleGAN model with bony structure constraints, which utilized Dixon fat and IP images as inputs, successfully generated sCT images of clinically acceptable quality for both bone and soft tissue. Among all tested models, this approach demonstrated the highest bone Dice similarity coefficient (DSC) of 0.88 and achieved the lowest mean absolute error (MAE) in both bone (145.2 HU) and the whole body (50.7 HU) compared to the planning CT. The dosimetric analysis of PTV and OARs showed that treatment planning recalculated based on sCT was highly comparable to that on reference CT images, with minimal dose distribution differences observed between the original CT‐based plan and the recalculated plan on sCT. These findings led us to start prospective evaluation of MROP. Figure 5 presents sCT and CT simulation images for three representative patients, along with quantitative HU comparisons. The sCT images demonstrated strong similarities to the CT images and HU difference maps were generated by subtracting sCT images from their corresponding CT images. These HU and dosimetric results were viewed favorably and enabled us to move forward with the prospective evaluation.

### 3.4 Prospective evaluation

Of the 12 prospective patients, 10 successfully completed treatment using the MROP workflow, including three SBRT (five fractions, total dose of 4500 cGy), and seven non‐SBRT cases (16–20 fractions, total dose of 6000–7000 cGy, five with nodal treatment). One patient was excluded due to inadequate bladder and rectal filling at time of MRI simulation. After this patient, we instituted the organ check scan. Another patient was excluded due to artifacts in the prostate in the sCT. Although density in the prostate could have been overridden, the physician opted to use the conventional CT simulation instead. Additionally, one patient's sCT showed low intensity in the bladder, creating the unreal appearance of air. In this case, we proceeded with MROP and manually overrode the bladder density. Following this case, we implemented an automatic density override step in the sCT generation process to ensure bladder ED was consistently set to water. The averaged HU difference, that is, the mean error (ME), instead of the MAE, accounting for both positive and negative values between sCT and CT across all MROP prospective patients, was −0.25 ± 5.90 HU for the entire body. The largest HU deviations were observed in bony structures, with a ME of 55.2 ± 24.3 HU. For dosimetric accuracy assessment, Table 2 summarizes the relative dose differences (absolute mean dose) between CT‐based and MROP‐based treatment plans for the targets and OARs. Among the 10 prostate treatment plans, three included simultaneous boost targets, and five involved the treatment of additional nodal volumes. The average dose differences across all planning target volumes (PTVs) were below 1%. Both positive and negative dose differences were observed between MROP‐based and CT‐based plans for the PTVs. The urethra exhibited the largest mean dose difference (15.2 cGy), while the sigmoid colon had the smallest mean dose difference (3.3 cGy). Error or approximation of HU number has been shown previously to have limited effect on dose calculation. Our results are consistent with these previous studies.^41^, ^42^, ^43^, ^44^

**TABLE 2:** Dose differences between CT and MROP based plan for targets (PTV) and organs at risk (OAR). For targets, % difference in Dmean and Dmax is used, for the OARs absolute difference is used.

			Dose difference (%)	
PTV	Cases	DVH point	Mean (SD)	(Min, Max)
Prostate	10	Dmean	0.1 (1.2)	(−1.0,3.0)
		Dmax	0.6 (1.5)	(−1.2, 3.6)
Boost	3	Dmean	−0.3 (0.5)	(−0.7,0.2)
		Dmax	0.4 (0.6)	(0.1, 1.1)
Nodes	5	Dmean	−0.6 (0.3)	(−0.9, −0.2)
		Dmax	−0.7 (0.4)	(−1.1, −0.2)
OAR			Dose difference (cGy)	
Bladder	10	Dmean	−11.8 (32.8)	(−99.2, 16.2)
Bowel	8	Dmean	−7 (21.9)	(−36.9, 33)
Rectum	10	Dmean	5.2 (20.4)	(−36.8, 28.6)
Sigmoid	8	Dmean	3.3 (18.6)	(−24.8, 41.1)
Urethra	10	Dmean	15.2 (48.3)	(31.7, 123.6)

### 3.5 Scheduling

After completing the prospective 10 patients, our department reviewed and approved the MROP workflow, and the backup CT was no longer scheduled. Prostate patients in our clinic were already receiving an MRI as part of our planning process, unless the patient was not an MRI candidate. By removing CT simulation, scheduling patients for MRI simulation became simpler as we no longer had to find a day where CT and MR were available back‐to‐back. This resulted in patient wait‐time from physician consult to simulation being reduced by 7.1 days in the first 6 months since clinical implementation compared to the 6 months prior, based on appointment data extracted from our electronic medical record system (Epic, Verona, WI). We originally added 1 day to our 5‐day planning timeline for sCT generation and review. We are now planning to remove that day as the process is smooth and the sCT generation and review can be in parallel with the contouring on the T2 MRI, which is used for contouring regardless of whether an sCT or CT is used for planning.

Before MROP, our average CT simulation time for prostate was 32 min, with the subsequent MR imaging in treatment position taking 49 min. For the MROP workflow, the MR simulation takes 59.5 min on average. MROP therefore provides an overall saving of 21 min on average. The timings reflect when the patient enters and leaves the simulation area, as recorded electronically by the therapists. Notably the time difference between an MR simulation with and without CT is only 10 min, which is the time required to create the immobilization, mark and photograph the patient.

### 3.6 MR‐Linac planning

After completing treatment for 10 prospective patients on the C‐arm Linac, we conducted a focused review of five MROP patient treated using the MRL (Unity, Elekta, Sweden). Key differences from the C‐arm workflow are highlighted. In the MRL process, the T2w images are used for both treatment planning and daily IGRT. The density of the T2w images is overridden on a per contour basis, with bone specifically contoured and assigned electron density from the sCT. For SBRT, a contour of the SBRT frame with a set density is added to the T2w images, along with the Unity couch and imaging coil. The couch index, where MR visible markers were placed on the patient or frame, was recorded during MR simulation to ensure accurate patient isocenter setup at Unity. FMs are not needed for MRL treatment. After observing no issues with these additional steps across the 5 patients, MROP was approved for clinical use with MRL in our department.

## 4 DISCUSSION

The MROP workflow has emerged as an effective strategy for prostate EBRT planning. Its clinical implementation can be challenging due to limitations in sCT accuracy and field of view, the difficulty of identifying FMs without CT, and the introduction of a significantly different process into clinic practice. We describe the development of an MROP program for prostate cancer treatment using both C‐arm Linac and MRL EBRT procedures. Our results demonstrate that the MROP workflow has been successfully optimized and implemented in clinical practice. Adopting MROP followed a structured approach, beginning with design, technical development, optimization, followed by validation, clinical go‐live, and continuous improvement.

Previous studies have utilized vendor software such as MRCAT (Philips) for sCT generation and have reported cases of errors or unrealistic reconstructions in overweight patients or those with metal implants, leading to workflow interruptions.^15^ Specifically, MRCAT is limited to a superior‐to‐inferior (SI) coverage of 240 mm and transverse FOV of 450 × 450 mm^2^, whereas the T1w Dixon sequence used in our protocol for sCT generation extends the SI coverage to 400 mm and the transverse FOV of 550 × 550 mm^2^. The extended FOV is crucial for treating cases involving both prostate and nodal volumes, as treatment planning requires coverage extending 5 cm above and below the target volumes. While commercial solutions offer convenience, a major limitation is the lack of flexibility for users to customize the workflow to their specific clinical practice. We also demonstrated the concept of hybridizing sCT with post‐processing via application programming interfaces (APIs) scripting. This approach can also allow adaptability of commercial solutions for site‐specific refinements, improving sCT quality, anatomical accuracy, and clinical usability. By integrating automated post‐processing, we addressed FM detection, frame insertion, and accuracy of bladder in sCT. The frame insertion concept used in this work is generalizable to other immobilization devices that users intend to visualize on MR images and incorporate into setup indexing. This flexibility allows the methodology to be adapted to other anatomical sites and immobilization techniques, further broadening the clinical applicability of MROP.

**FIGURE 6:**
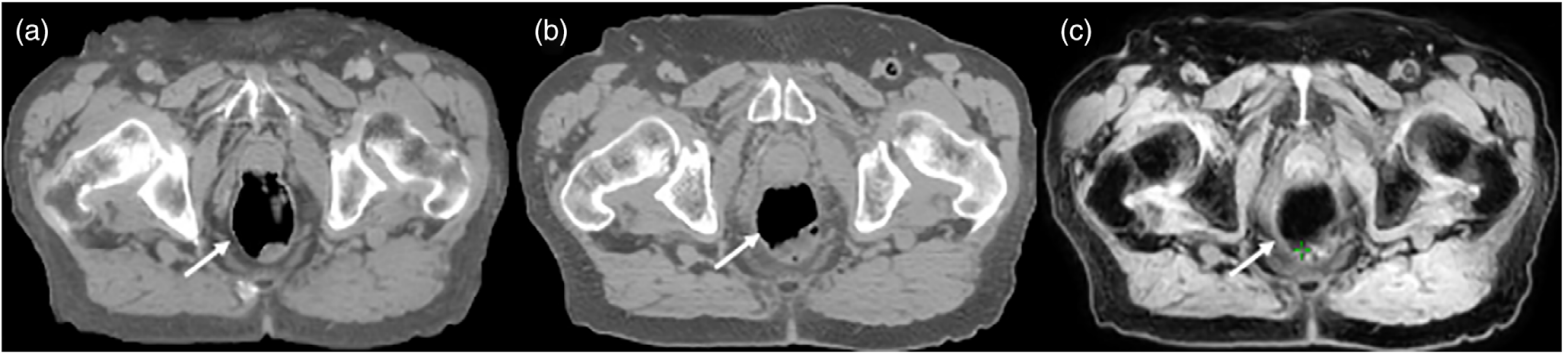
A prospective patient case with the arrow highlighting signal voids and susceptibility artifacts extending from the rectum into the prostate region on the sCT (a), attributed to excessive gas in the rectum. In contrast, our improved sCT model (b) demonstrated accurate conversion from the T1w water image (c) to sCT (b).

The rapid development of deep learning technology in image‐to‐image translation has enabled novel approaches for MR‐to‐sCT conversion.^45^ Our sCT generation algorithm employed a multichannel CycleGAN model using fat and in‐phase images acquired from a T1w Dixon MRI sequence to enhance the accuracy of bony structure representation. Among 12 prospective patients, two exhibited significant artifacts on sCT. One case presented contrast‐enhanced spots inside the prostate, which are not typically seen on CT; further investigation revealed that these corresponded to large dark spots on MRI caused by FMs with a large diameter. The second case displayed signal voids and susceptibility artifacts extending into the prostate region on the sCT, attributed to excessive gas in the rectum (Figure 6a). Additionally, in some cases, inhomogeneous signal intensity in the bladder resulted in small patches on the sCT incorrectly assigned to air HU value. To further improve the sCT accuracy and workflow, we refined our sCT model by incorporating bladder segmentation as a guidance, ensuring a more uniform HU distribution in the generated sCT and avoided gas generation beyond the rectum. Any remaining issues can be corrected by manual density overrides during planning in the sCT. An example of the improved sCT for a patient is shown in Figure 6b. Since instituting the model enhancements, only eight out of approximately 350 patients required manual density overrides of the sCT before treatment planning.

For FM detection, manual cross‐verification by a dosimetrist or physicist is always required. Due to the long acquisition time for each MRI sequence (3–5 min), the positions of the prostate and OARs may shift between sequences due to bladder filling or rectal deformation. This can lead to FM misalignment between the internal marker T2*w GRE images, T1w Dixon images, and T2w 3D images. To address this, we developed a quality assurance (QA) process. In this process, automatically detected FMs are first cross‐validated with the signal void locations on T1w Dixon (water) images, then transferred onto T2w 3D images for a final check. This cross‐verification helps prevent false FM detections, and minor adjustments may be necessary due to anatomical changes. The FM locations are finalized on T2w 3D images, as these serve as the primary images for contouring. This FM detection strategy is generalizable. The key is to streamline the process for users, and APIs offer a practical solution. For instance, if users wish to enhance the sCT provided by a vendor, they could integrate a post‐processing API script to embed the markers (e.g., burn in the markers at high HUs).

One key limitation of the MROP workflow is the long scan time, which can lead to organ or FM movement between sequences, potentially affecting image alignment. Future technological advancements, such as accelerated MRI acquisition or the use of MRI fingerprinting for multi‐contrast imaging, may help mitigate this issue. Other limitations include large patient size, which may exceed coil coverage, resulting in an incomplete FOV and compromising body contour accuracy. Metal artifacts are another concern, as they can degrade image quality and interfere with beam arrangement in treatment planning. While metal artifact reduction techniques can improve image quality, they often require longer acquisition times, further extending the overall scan duration. In addition to the technical and logistical challenges discussed, there are several practical clinical issues that warrant mention. Another challenge we encountered relates to image quality degradation in a small subset of patients (*n* < 5) who presented with excessive bowel gas during MR simulation. In these cases, gas‐induced artifacts significantly impaired the ability to clearly visualize key pelvic structures, including the prostate peripheral zone and rectum. This limitation underscores the importance of bowel preparation protocols and possibly the need for real‐time image quality checks prior to simulation.

One current limitation is the variability and placement of FMs used across specialties. For instance, urologists at our institution have recently adopted a different type of FM that is less conspicuous on our current MRI sequences. This has introduced difficulties in accurately localizing the markers, particularly when planning treatment on CT‐based machines. To address this, it maybe be necessary to update or develop new MRI sequences to enhance FM visualization and improve image registration accuracy. For non‐SBRT patients, cone‐beam CT images acquired at the time of first fraction of treatment can be used to verify and correct marker locations. For SBRT patients, a CT scan prior to finishing planning, with or without immobilization, can be used to refine the marker location.

Table 3 summarizes the exceptions from the MROP workflow since it became clinical at our institution. It shows that about 7% of patients may be ineligible for MROP. Additional steps to the workflow are required in around another 7% of patients. Note, the patient implant number comes from^38^ as we do not track patients that were never offered MROP because of an implant.

**TABLE 3:** Patients with workflow exceptions. The upper three issues prevent the patient from using the MROP workflow. The lower three require additional, manual steps.

Workflow exception	Patients
Patient Implant	5%
Patient diameter > 49″	1%
Bowel gas artifacts	1%
Density override required	2%
Fiducial marker movement	2%
Additional CBCT/CT	3%

We reported an average displacement of 2.6 mm between the FM center in MR and CT. This is an upper limit on the error in location of the FM in MR versus its true position relative to the prostate, which is what we wish to align during image guidance. The overall error may be lower depending on the accuracy of CT FM location. This displacement would comprise the bulk of the PTV margin for SBRT cases (3 mm at our institution), which is meant to cover both setup and motion errors. The potential increase in FM uncertainty relative to CT, is balanced by the higher accuracy of the MR prostate delineation. The MR‐linac, which uses the prostate directly for alignment avoids these uncertainties and could be considered for reduced margins.

The use of T1w Dixon sequences is excellent for sCT generation and can be extended to other body sites. As with the prostate, there will be technical as well as workflow issues. Each site will require its own refinements and additions to the sCT generation, for example lung in thoracic sites. Workflow issues will largely remain common with prostate MROP, including patient selection, creating immobilization at the MR, patient marking and anatomy movement between MR sequence acquisition. This experience with prostate MROP workflow can be useful for implementing MROP on other body sites.

## 5 CONCLUSION

We successfully developed an MROP workflow for prostate and prostate plus nodes patients, integrating technical innovations and workflow optimization, supported by comprehensive retrospective and prospective patient validations. The proposed MROP workflows for X‐ray‐based IGRT on C‐arm Linacs and MRIgRT on MRL enhance clinical efficiency by reducing imaging dose, patient time, and simulation‐related costs, while also eliminating CT‐MRI registration errors. Since its clinical go‐live in June 2023, the physician, physicist, dosimetrist, and therapist teams have continued to collaborate diligently to refine the techniques and workflow by identifying and troubleshooting issues in daily clinic practice. To date, we have successfully treated over 500 patients on both C‐arm Linac and MRL systems. We are now working on expanding the sCT generation to all body sites.

## AUTHOR CONTRIBUTIONS

Conceptualization of study (Jie Deng, Raquibul Hannan, Neil Desai, Daniel Yang, Aurelie Garant, Mu‐Han. Lin, Steve Jiang, Andrew Godley), execution of study (Jie Deng, Raquibul Hannan, Neil Desai, Daniel Yang, Aurelie Garant, Andrew Godley, Mu‐Han. Lin, Viktor Iakovenko), Data curation and analysis (Xiao Liang, Boyu Meng), Software (Xiao Liang, Junjie Wu, Weiguo Lu, Yang K. Park), Writing (Jie Deng, Xiao Liang, Viktor Iakovenko, Boyu Meng, Mu‐Han. Lin, Andrew Godley), Review (Jie Deng, Xiao Liang, Aurelie Garant, Andrew Godley).

## CONFLICT OF INTEREST STATEMENT

The authors declare no conflicts of interest.
